# 

**Published:** 1902-07-15

**Authors:** 


### CONTENTS.

President's Address, - - - *By C. H. Oakman* 325

Pyorrhea Alveolaris, - - *By E. B. Newell, D.D.S.* 331

Reminiscences and Things, *By J. S. Cassidy, D.D.S.* 354

Soldering Hint, - - *By L. C. Taylor, D.D.S.* 359

The New Ohio Dental Law, (*House Bill No. 151*) - 360

EDITORIAL, - - - - - - - - - 366

ANNOUNCEMENTS, - - - - - - - - 368

BIBLIOGRAPHY, - - - - - - - - 369

INDENTS, - - - - - - - - - 370

